# Inferior subluxation of humeral head after osteosynthesis for greater tuberosity fracture

**DOI:** 10.1186/s13018-022-03379-9

**Published:** 2022-11-03

**Authors:** Ryogo Furuhata, Atsushi Tanji, Satoshi Oki, Yusaku Kamata

**Affiliations:** 1 Department of Orthopaedic Surgery, Ashikaga Red Cross Hospital, 284-1 Yobe-Cho, Ashikaga-Shi, Tochigi 326-0843 Japan; 2 Department of Orthopaedic Surgery, Saiseikai Utsunomiya Hospital, Utsunomiya-Shi, Tochigi Japan; 3 Department of Orthopaedic Surgery, National Hospital Organization Tokyo Medical Center, Meguro-Ku, Tokyo, Japan

**Keywords:** Humeral head, Inferior subluxation, Greater tuberosity fracture, Osteosynthesis, Outcome

## Abstract

**Background:**

Inferior subluxation of the humeral head is frequently observed immediately after surgery for proximal humerus fractures; however, the incidence and risk factors of inferior subluxation after osteosynthesis for isolated greater tuberosity fractures remain unsolved. Additionally, the postoperative course of inferior subluxation has not been elucidated. The purpose of the present study is to identify the predictors for the occurrence of postoperative inferior subluxation by multivariate analysis and investigate the postoperative shift of inferior subluxation and its effect on surgical outcomes.

**Methods:**

We retrospectively identified 68 patients who underwent surgery for isolated greater tuberosity fractures. The dependent variable was the inferior subluxation at 1 week postoperatively. The explanatory variables were age, sex, affected side of the shoulder, body mass index, history of smoking, local osteoporosis, time period to surgery, axillary nerve injury, inferior subluxation before surgery, fracture dislocation, surgical approach, surgical method, operative time, amount of blood loss, and postoperative drainage. Baseline variables that were statistically significant in the univariate analyses were included in the logistic regression analysis. The patients were further categorized into two groups according to the presence of inferior shoulder subluxation exhibited 1 week postoperatively: patients with inferior subluxation (+ IS group) and patients without inferior subluxation (− IS group). We compared the incidence of postoperative complications between the two groups.

**Results:**

Of 68 patients, 17 (25.0%) had inferior shoulder subluxation observed 1 week postoperatively. Multivariate analysis showed that long operative time was a risk factor for postoperative subluxation (odds ratio = 1.03; *P* = 0.030). In all cases, inferior subluxation disappeared within 3 months of surgery. No significant difference in complication rate was observed between the + IS and − IS groups.

**Conclusions:**

The present study provides novel information regarding postoperative inferior subluxation of fractures of the greater tuberosity. Inferior subluxation occurred in 25% of patients immediately after surgery. Long operative time contributes to the onset of postoperative inferior subluxation; however, this was temporary in all cases and had no significant effect on the surgical outcomes.

*Level of Evidence*: Level III.

## Background

Inferior subluxation of the humeral head is frequently experienced immediately after osteosynthesis for proximal humerus fractures and occurs in 31–42% of patients at 1–2 weeks after surgery [1, 2]. Most cases are transitory and improve with time; however, persistent inferior shoulder subluxation observed 1 year postoperatively is associated with screw articular surface perforation and low Constant score [3]. Therefore, it is important to identify the risk factors of postoperative subluxation and investigate its postoperative course.

The causes of inferior subluxation after acute shoulder trauma include muscle fatigue, such as that of the deltoid muscle [4], atony of deltoid and rotator cuff muscles [1, 3, 5], loss of negative intraarticular pressure of glenohumeral joint [1], peripheral nerve injury [6], and capsular injury [7]. However, there is limited evidence on the factors affecting inferior subluxation exhibited immediately after osteosynthesis for proximal humerus fractures [2, 3]. In particular, the incidence of inferior subluxation after an isolated fracture of the greater tuberosity and the factors affecting its onset remain unknown. Furthermore, the postoperative changes of inferior subluxation, postoperative progress, and the effect of postoperative subluxation on surgical outcomes have not been previously reported.

The aims of the present study were: (1) to identify the incidence of inferior subluxation immediately after osteosynthesis for isolated fracture of the greater tuberosity, and the factors that affect the incidence by multivariate analysis, and (2) to investigate the postoperative course of subluxation and to analyze the influence of postoperative subluxation on surgical outcomes. This study was approved by the Independent Ethics Committee of our hospitals.

## Methods

### Study design and patients

This was a retrospective study including patients who underwent osteosynthesis for a fracture of the greater tuberosity at three municipal general hospitals between 2008 and 2021. We included adult patients who underwent surgery for isolated greater tuberosity fracture diagnosed by plain radiographs and computed tomography (CT). Several previous studies have recommended that a superior displacement of the greater tuberosity of ≥ 5 mm is an indication for surgery of the greater tuberosity fracture [8, 9] as it is thought to cause abnormal shoulder mechanics during shoulder abduction [10] and subacromial impingement [8, 11]. Therefore, the indications for surgery for greater tuberosity fractures at the institution where this study was performed were patients who could undergo general anesthesia and had a superior displacement of greater than 5 mm. We determined a superior displacement when the superior margin of the greater tuberosity fragment was ≥ 5 mm superior to the superior margin of the articular fragment of the humeral head on the anteroposterior view of plain radiography or the coronal view of CT, regardless of the fracture pattern. We excluded patients with other fractures complicating the affected upper extremity, history of surgery involving the affected upper extremity, and paralysis of the affected upper extremity due to cerebral infarction or other causes, and patients who underwent osteosynthesis using intramedullary nail.

### Surgical procedure

Eleven orthopedic surgeons performed surgery. In all cases, surgery was performed in the beach-chair position under general anesthesia. Osteosynthesis was performed using the delto-pectoral or deltoid split approaches using plates in 24 patients, cannulated cancellous screw (CCS) in 16 patients, transosseous wiring or suture in 15 patients, suture-bridge technique in 8 patients, and tension band wiring (TBW) in 5 patients, at the discretion of the surgeon. The implant used for plate fixation was the PHILOS® plate (Depuy Synthes, Oberdorf, Switzerland), LCP® plate (Depuy Synthes, Oberdorf, Switzerland), or MODE® plate (MDM, Tokyo, Japan). The implant used for CCS fixation was the ACE® (Zimmer Biomet, Warsaw, IN, USA) or Asnis® III cannulated screw system (Stryker, Kalamazoo, MI, USA). For transosseous wiring or suture, a surgical wire or FiberWire® (Arthrex, Naples, FL, USA) was fastened through the rotator cuff and the bone hole created distally in the humeral fragment. For the suture-bridge procedure, suture anchors were inserted proximally and distally to the fracture site and the bone fragments were reduced and fixed. Healix Advance™ BR anchor (Mitek, Raynham, MA, USA), JuggerKnot® anchor (Zimmer Biomet, Warsaw, IN, USA), and Quattro® Link Knotless Anchor (Zimmer Biomet, Warsaw, IN, USA) were used. TBW was performed using Kirschner wires and surgical wires, AI-Wiring system (Aimedic MMT, Tokyo, Japan) or RING PIN system (Nakashima Medical, Okayama, Japan). In this study, the mean operative time and blood loss for osteosynthesis performed in this study were 100.5 ± 32.5 min and 65.5 ± 98.1 g, respectively. In eight cases, drainage was performed by inserting an SB VAC™ (Sumitomo Bakelite, Tokyo, Japan) into the fracture site for 2 days after osteosynthesis. Immobilization of the arm was achieved by sling fixation postoperatively for 1–3 weeks after which passive range of motion (ROM) exercises were initiated. Active ROM exercises were initiated 4–6 weeks after surgery. Patients did not undergo additional fixation periods even when inferior subluxation occurred postoperatively.

### Radiographic evaluation of inferior subluxation of humeral head

**Fig. 1:**
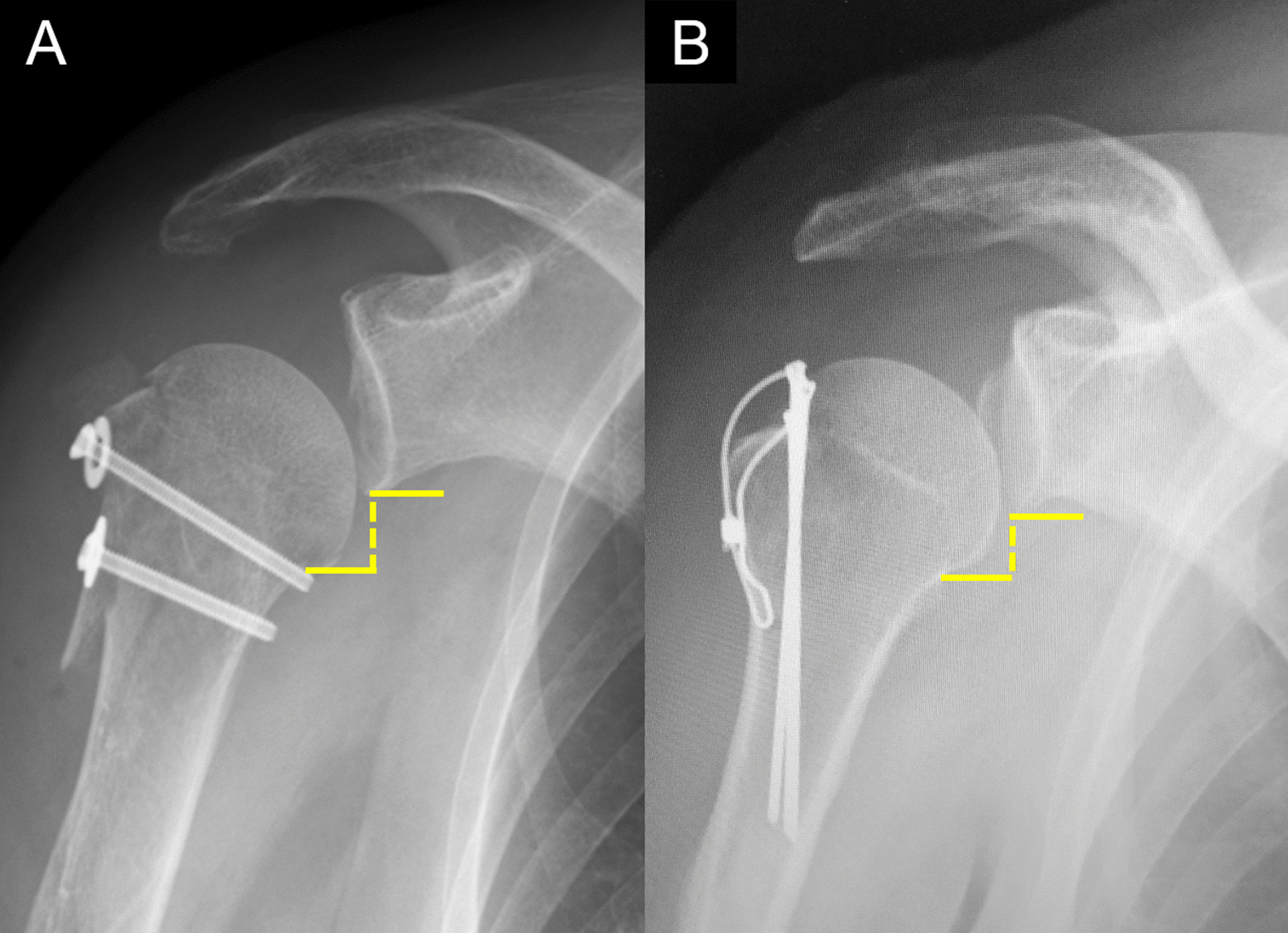
Radiographic assessment of inferior subluxation of the humeral head. A distance of ≥ 1 cm between the humeral anatomical neck level and the glenoid inferior edge level was defined as the presence of humeral head inferior subluxation. Postoperative radiograph images after surgery using a cannulated cancellous screw (**A**) or tension band wiring (**B**)

Various methods of humeral head inferior subluxation have been previously reported [3, 12, 13]. Carbone et al. define inferior subluxation as a distance of ≥ 1 cm between the humeral anatomical neck level and the glenoid inferior edge level [3]. We adopted this method for this study because a good intra- and excellent inter-rater reliability was reported [3]. Based on the previous studies [3, 13], plain radiographs of the shoulder in the upright position taken before surgery and at 1 week, 1 month, 3 months, and 6 months postoperatively were evaluated by one examiner. Inferior subluxation on the plain radiograph taken 1 week postoperatively was defined as inferior subluxation immediately after osteosynthesis, as previously described [2] (Fig. 1).

The patients were further divided into two groups according to the presence of inferior subluxation at 1 week postoperatively: patients with inferior subluxation (+ IS group) and patients without inferior subluxation (−  IS group).

### Outcome measures

Multivariate analysis was performed to clarify the factors affecting postoperative subluxation, and inferior subluxation on plain radiograph at 1 week postoperatively was used as the dependent variable. The explanatory variables were age, sex, affected side of the shoulder, body mass index (BMI), history of smoking, local osteoporosis, time period from injury to surgery, preoperative axillary nerve injury, fracture dislocation, preoperative inferior subluxation, surgical approach (delto-pectoral/deltoid split approach), surgical method (plate, CCS, transosseous wiring, suture-bridge technique, TBW), operative time, amount of blood loss, and drainage after surgery. Local osteoporosis was assessed by measuring the average cortical thickness at two points of the humerus, and an average proximal humeral cortical thickness of ˂ 6 mm was defined as local osteoporosis as previously reported [14]. Axillary nerve injury was assessed using clinical notes on numbness of the axillary nerve region.

Postoperative outcomes were postoperative complication rate (delayed bone union, nonunion, infection, screw perforation into the joint, fixation failure) and ROM (elevation and external rotation [ER] at side) at 6 months after surgery. A single evaluator, who was blinded on the results of postoperative inferior subluxation, investigated the postoperative complications based on the clinical notes and plain radiographic images. Delayed union was defined as a lack of bone bridging at 6 months postoperatively. We defined fixation failure as a residual displacement of the greater tuberosity fragment of ≥ 5 mm. Postoperative ROM was assessed by the surgeon who performed osteosynthesis or the occupational therapist. We compared the postoperative outcomes between the + IS and −  IS groups during a follow-up period ≥ 6 months.

### Statistical analysis

All statistical analysis was conducted using SPSS software (version 27.0*, IBM, Armonk, NY, USA). In univariate analyses, we used the Mann–Whitney U test to compare the average of continuous values (age, BMI, time from injury to surgery, operative time, and blood loss). We used Fischer’s exact test to compare the proportions (sex, side of injury, smoking, local osteoporosis, preoperative axillary nerve injury, fracture dislocation, preoperative inferior subluxation, surgical approach, surgical method, and postoperative drainage). Baseline variables with *P* < 0.05 in univariate analysis were included in the logistic regression analysis to clarify the independent predictive factors of inferior subluxation. The regression model fit was estimated by the Hosmer–Lemeshow goodness-of-fit test. On the examination of the effect of subluxation on surgical outcomes, the Mann–Whitney U test was used to compare the average of ROM and Fischer’s exact test was used to compare the complication rate. *P* < 0.05 was considered statistically significant.

## Results

In total, 68 patients met the inclusion and exclusion criteria. The patient mean age was 61.4 ± 15.7 (range 28–92) years; 42 were women, and 26 were men. The injury was on the right and on the left side in 37 and 31 patients, respectively. Preoperative axillary nerve injury and humeral head inferior subluxation were observed in 11 (16.2%) and seven (10.3%) patients, respectively. The injury consisted of fracture dislocation in 31 patients (45.6%).

Of the 68 patients, 17 (25.0%) exhibited inferior subluxation at 1 week postoperatively; therefore, the + IS and −  IS groups included 17 and 51 patients, respectively.　The results of the univariate analysis revealed that surgical method (plate) (*P* = 0.038), longer operative time (*P* = 0.002), and higher blood loss (*P* = 0.048) were significantly associated with the incidence of inferior subluxation immediately after osteosynthesis. Multivariate analyses revealed that longer operative time (odds ratio = 1.03; 95% confidence interval = 1.00–1.05; *P* = 0.030) was a risk factor for postoperative subluxation. The Hosmer–Lemeshow goodness-of-fit test showed no significant difference from good model fit (*P* = 0.525) (Table 1).

**Table 1:** Univariate and multivariate predictors of inferior subluxation at 1 week after surgery

Variables	Univariate predictors			Multivariate predictors	
	+ IS group (*N* = 17)	− IS group (*N* = 51)	*P*-value	Odds ratio [95% CI]	*P*-value
Age (years)	60.4 [52.6–68.2]	61.7 [57.4–66.0]	0.686	–	–
Sex (female/male)	11/6	31/20	1	–	–
Affected side (right/left)	12/5	25/26	0.163	–	–
BMI	24.2 [22.4–26.0]	24.0 [22.8–25.1]	0.766	–	–
Smoking	6	16	0.772	–	–
Local osteoporosis	5	17	1	–	–
Time from injury to surgery (days)	8.1 [5.5–11.7]	9.7 [7.3–12.2]	0.599	–	–
Preoperative axillary nerve injury	2	9	0.718	–	–
Dislocation fracture	9	22	0.578	–	–
Preoperative inferior subluxation	3	4	0.355	–	–
Surgical approach (Delto-pectoral/Deltoid split)	12/5	23/28	0.094	–	–
*Surgical method*					
Plate	10	14	0.038*	1.77 [0.47–6.65]	0.397
CCS	3	13	0.743	–	–
TO	4	11	1	–	–
Suture-bridge	0	8	0.186	–	–
TBW	1	4	1	–	–
Operative time (minutes)	121 [109–133]	94 [85–102]	0.002*	1.03 [1.00–1.05]	0.030*
Blood loss (g)	83 [49–116]	60 [31–89]	0.048*	1.00 [1.00–1.01]	0.937
Postoperative drainage	3	5	0.402	–	–

*CI* Confidence interval, *BMI* Body mass index, *CCS* Cannulated cancellous screw, *TO* Transosseous wire or suture, *TBW* Tension band wiring

Numbers in square brackets indicate 95% CI

^*^P < 0.05,

**Fig. 2:**
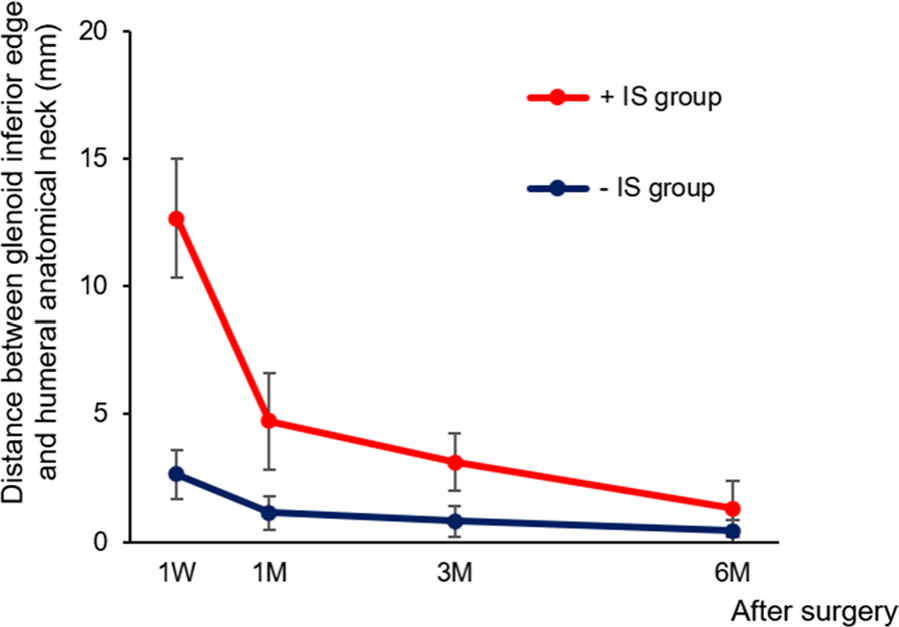
Postoperative course of postoperative inferior subluxation of the humeral head. The blots show the distance between the humeral anatomical neck level and glenoid inferior edge level in the + IS (red) and – IS groups (blue) at 1 week, 1 month, 3 months, and 6 months after surgery for greater tuberosity fracture. Error bar represents a 95% confidence interval. M; month, W; week

Figure 2 shows the postoperative course of inferior subluxation of the + IS and –IS groups. This graph presents the postoperatively narrowing distance between the humeral anatomical neck level and glenoid inferior edge level with time in both groups. Inferior subluxation persisted at 1 month postoperatively in two patients in the + IS group, although it disappeared in all patients at 3 and 6 postoperative months. No patients in the –IS group developed a new inferior subluxation within 6 months after osteosynthesis.

On examination of the effect of subluxation immediately after osteosynthesis on surgical outcomes, we identified 60 patients with a follow-up period ≥ 6 months postoperatively. Two patients in the + IS group and six patients in the −  IS group were excluded because of insufficient follow-up. The data for ROM were missing for elevation in 12 patients and for ER in 23 patients.

No significant difference in complication rate was noted between the + IS and −  IS groups (Table 2). Two patients with delayed union achieved union within the 1-year postoperative follow-up period. None of the patients with fixation failure required reoperation. Additionally, no significant difference was noted between the + IS group and −  IS group in ROM of elevation (142° [131–149°] vs. 140° [131–149°], respectively, *P* = 0.953) and ER (41° [30–52°] vs. 52° [46–59°], respectively, *P* = 0.129) at 6 months postoperatively.

**Table 2:** Postoperative complication (+ IS group vs. –IS group)

Complications *N* (%)	+ IS group (*N* = 15)	− IS group (*N* = 45)	*P*-value
Delayed union	2 (13.3%)	1 (2.2%)	0.151
Nonunion	0 (0%)	0 (0%)	1
Infection	0 (0%)	0 (0%)	1
Screw perforation	0 (0%)	0 (0%)	1
Fixation failure	1 (6.7%)	5 (11.1%)	1

## Discussion

In the present study, we performed a multivariate analysis to clarify the factors affecting the onset of inferior subluxation immediately after osteosynthesis for isolated greater tuberosity fractures and investigated the postoperative prognosis of inferior subluxation. Two important clinical observations should be noted.

First, the results of the multivariate analysis revealed that operative time significantly affected the incidence of inferior subluxation at 1 week postoperatively for isolated fracture of the greater tuberosity. This finding is consistent with a previous study investigating postoperative inferior subluxation of humeral neck fracture or 3-part proximal humerus fracture [2]. The mechanism of how operative time affects inferior subluxation immediately after surgery remains unknown; however, retraction of the muscles attached to the humerus such as the deltoid muscle or rotator cuff for a long period during surgery may cause muscle fatigue or atony, or a long operative time may affect peripheral nerve traction and compression, leading to postoperative inferior subluxation. Preoperative inferior subluxation was also shown to be a significant risk factor for inferior subluxation immediately after surgery [2]; however, preoperative inferior subluxation was not identified as a significant factor in the present study. This difference may be ascribed to the fact that 25% of patients in the reported study had preoperative inferior subluxation [2], while this was observed in a mere 10% of the patients in this study with isolated greater tuberosity fractures. Another study reported significant associations between inferior subluxation at 3 months postoperatively and older age, female sex, obesity, and screw joint perforation [3]. Although female sex and high BMI were observed in a slightly higher number of patients with postoperative inferior subluxation in the univariate analysis, these differences were not significant. This gap may be accounted for by the fact that the present analysis focused on patients at an earlier stage after surgery (at 1 week postoperatively), so surgery-related factors may have had a greater influence on inferior subluxation than patient demographics such as age, sex, and obesity.

Second, inferior subluxation was observed in 25% of patients immediately after surgery for a greater tuberosity fracture; however, this improved in all cases within 3 months of surgery and the presence of inferior subluxation just after surgery had no significant influence on surgical outcome at 6 months after surgery. The reported incidence of inferior subluxation observed immediately after osteosynthesis for all proximal humerus fractures is 31–42%, which exceeds the incidence of subluxation after osteosynthesis for the isolated greater tuberosity fractures reported in this study. Moreover, while inferior subluxation has been reported to persist more than 6 months after surgery in 2.8–4.6% of patients [2, 3], it improved within 3 months postoperatively in all patients in this study. These findings suggest that compared to proximal humerus fractures, isolated fractures of the greater tuberosity are associated with a lower incidence and earlier recovery of inferior subluxation observed immediately after surgery. This difference may also be explained by the lower invasiveness of surgery for fractures of the greater tuberosity compared to surgery for proximal humerus fractures. This is consistent with the shorter operative time and smaller blood loss observed in this study compared to that in previous reports [2]. Additionally, while persisting inferior subluxation one year after surgery for proximal humerus fracture is related to complication such as screw joint perforation [3], this complication is rarer following surgery for isolated fracture of the greater tuberosity, thereby potentially contributing to the improvement in inferior subluxation in all patients. Despite there being significantly more patients with longer operative times and greater blood loss in the + IS group than in the −  IS group, no significant between-group differences were noted in complication rate or ROM at 6 months postoperatively. These results were consistent with those of previous studies on proximal humerus fractures that demonstrated that postoperative inferior subluxation had no significant influence on the surgical outcome [2, 3].

The incidence of inferior subluxation 1 week after osteosynthesis for proximal humerus fractures has been reported to be 31% [2]; therefore, based on a power analysis assuming a 30% rate of postoperative inferior subluxation, approximately 100 patients would be required to show a 50% difference in the incidence of postoperative inferior subluxation in this study. Based on this, the study may have been influenced by factors that could not be detected (β-error) in the univariate analysis that examined the predictors of postoperative inferior subluxation. However, the number of fractures were relatively large in this clinical study on 68 patients, while the majority of reports on surgical outcomes of greater tuberosity fractures had a sample size of 50 or less [9, 15]. This can be a strength of this study.

However, there are several limitations in this study. The first limitation is the observational nature of this study, which may have overlooked residual, unmeasured confounders that may also play a role in the difference between the groups. For example, the surgical procedures were performed by 11 orthopedic surgeons in this study, but we did not analyze the effects of the skills of surgeons and their assistants. Moreover, the choice of implant type and surgical technique should be consistent based on the size or comminution of fracture fragment or bone density; however, the fact that the choice of implants depended on each surgeon’s preference could be a limitation of this work. Second, this survey did not include a questionnaire, so we could not measure additional objective functional outcomes. Third, there are missing data for the postoperative ROM, which may have resulted in inadequate assessment of ROM.

## Conclusions

The present study provides novel information regarding postoperative inferior subluxation of greater tuberosity fractures. Inferior subluxation occurred immediately after surgery in 25% of patients. Long operative time was associated with postoperative inferior subluxation; however, this was temporary in all cases and had no significant effect on the surgical outcomes.

## Data Availability

Data that support the findings of this study are available from the corresponding author on reasonable request.

## Abbreviations

CT: Computed tomography
CCS: Cannulated cancellous screw
TBW: Tension band wiring
ROM: Range of motion
IS: Inferior subluxation
ER: External rotation
